# Combined cumulative sum (CUSUM) and chronological environmental analysis as a tool to improve the learning environment for linear-probe endobronchial ultrasound-guided transbronchial needle aspiration (EBUS-TBNA) trainees: a pilot study

**DOI:** 10.1186/s12890-017-0375-9

**Published:** 2017-02-07

**Authors:** Yasuhiro Norisue, Yasuharu Tokuda, Mayrol Juarez, Ryo Uchimido, Shigeki Fujitani, David A. Stoeckel

**Affiliations:** 1 Department of Pulmonary and Critical Care Medicine, Tokyo Bay Urayasu-Ichikawa Medical Center, 3-4-32 Toudaijima, Urayasushi, Chiba 279-0001 Japan; 2 Japan Community Healthcare Organization, 3-22-12, Takanawa, Tokyo, 108-0074 Japan; 3 Mercy Hospital, 1235 E Cherokee St, Springfield, MO 65804 USA; 4 Beth Israel Deaconess Medical Center, 330 Brookline Ave, Boston, MA 02215 USA; 5 Department of Emergency and Critical Care Medicine, St. Marianna University Hospital, 2-16-1, Miyamae, Kawasaki, Kanagawa Japan; 6 Division of Pulmonary, Critical Care and Sleep Medicine, Saint Louis University, 1402S. Grand Avenue, Saint Louis, MO 63104-1004 USA

**Keywords:** CUSUM analysis, Quality improvement, Endobronchial ultrasound-guided transbronchial needle aspiration, EBUS-TBNA, CUSUM analysis, Quality improvement

## Abstract

**Background:**

Cumulative sum (CUSUM) analysis can be used to continuously monitor the performance of an individual or process and detect deviations from a preset or standard level of achievement. However, no previous study has evaluated the utility of CUSUM analysis in facilitating timely environmental assessment and interventions to improve performance of linear-probe endobronchial ultrasound-guided transbronchial needle aspiration (EBUS-TBNA). The aim of this study was to evaluate the usefulness of combined CUSUM and chronological environmental analysis as a tool to improve the learning environment for EBUS-TBNA trainees.

**Methods:**

This study was an observational chart review. To determine if performance was acceptable, CUSUM analysis was used to track procedural outcomes of trainees in EBUS-TBNA. To investigate chronological changes in the learning environment, multivariate logistic regression analysis was used to compare several indices before and after time points when significant changes occurred in proficiency.

**Results:**

Presence of an additional attending bronchoscopist was inversely associated with nonproficiency (odds ratio, 0.117; 95% confidence interval, 0–0.749; *P* = 0.019). Other factors, including presence of an on-site cytopathologist and dose of sedatives used, were not significantly associated with duration of nonproficiency.

**Conclusions:**

Combined CUSUM and chronological environmental analysis may be useful in hastening interventions that improve performance of EBUS-TBNA.

## Background

Cumulative sum (CUSUM) analysis is a method of monitoring the performance of an individual or process and detect deviations from a preset or standard level of achievement [1, 2]. It has been used in medical disciplines, particularly in monitoring and characterizing trainee learning curves. In theory, CUSUM analysis is an ideal tool to improve the quality of medical procedures, as it can alert observers to performance declines or improvements among workers, thus facilitating timely assessment of the learning environment. However, no previous study has examined the utility of combining CUSUM analysis with assessment of the learning environment for medical procedures.

Linear-probe endobronchial ultrasound-guided transbronchial needle aspiration (EBUS-TBNA) is a bronchoscopic technique that uses ultrasound to visualize and guide sampling of mediastinal and hilar lymph nodes [3, 4]. Kemp et al. used CUSUM analysis to plot learning curves for EBUS-TBNA and found that the curves varied widely among bronchoscopists [5]. Differences in learning speed among bronchoscopists at the same institution are mostly attributable to variation in their individual characteristics. However, if a bronchoscopist has an unexpected important change in proficiency in CUSUM analysis, investigating the learning environment and current performance characteristics of the bronchoscopist could help in implementing appropriate early interventions.

This study investigated whether CUSUM analysis could be used to monitor individual and institutional proficiency in linear EBUS-TBNA and improve the quality of the learning environment for EBUS-TBNA. CUSUM analysis was used to monitor EBUS-TBNA performance of bronchoscopists during training and investigate the relationship between change in performance and changes in the characteristics of the learning environment in the bronchoscopy suite.

## Methods

### Study design and setting

Linear-probe EBUS-TBNA bronchoscopy has been performed since March 2008 at our institution, the Saint Louis University Hospital. All attending bronchoscopists had more than 5 years of experience in bronchoscopy without EBUS and had received less than 1 week of EBUS-TBNA training before EBUS-TBNA was initiated at our center. We analyzed data from 131 patients evaluated from March 2008 to September 2010 by 3 bronchoscopists (bronchoscopist A: 47 patients with 84 lymph nodes sampled; bronchoscopist B: 50 patients with 89 lymph nodes sample; and bronchoscopist C: 34 patients with 49 lymph nodes sampled). All procedures utilized 22G needles.

Charts and procedure notes were examined to collect information on patient demographics, EBUS-TBNA indications, the procedures (date, operator(s), sampled lymph nodes, complications), final cytology results of transbronchial needle aspiration for each node, pathology results for mediastinoscopy, CT-guided lymph node biopsy, video-assisted thoracic surgery and thoracotomy, findings of subsequent imaging studies, and final clinical diagnosis.

Information on bronchoscopy suite conditions that was available retrospectively included the name of the main attending bronchoscopist (resident and fellow physicians were not included in the analysis), the names of attending bronchoscopists who assisted the main bronchoscopist, the doses of sedatives and opiates used during each procedure, and the presence of cytopathologists for rapid on-site evaluation of specimens. Two bronchoscopy nurses were present for all procedures. This observational chart review was approved by the Institutional Review Board at Saint Louis University.

Kemp et al. defined success on EBUS-TBNA as a true positive or true negative for malignancy, and failure as a false positive or false negative for malignancy. However, a procedure that successfully samples a lymph node but yields a false negative should not be defined as operator failure, as it represents a limitation of the EBUS-TBNA technique itself rather than a lack of operator skill. We believe that procedure failure should instead be defined as absence of lymphocytes, malignant cells, or granuloma in a specimen. In other words, a procedure is successful when the lymph node is adequately visualized and sampled, as determined by the presence of diagnostic cells or lymphocytes in the sample.

### Statistical analysis

The primary outcome was sampling failure or success. Each sampled lymph node was used as the unit for determining success or failure. A lymph node sampling was defined as successful if 1 or more cytology slides showed lymphocytes, granulomas, or malignant cells.

CUSUM analysis has been used to continuously monitor procedural outcomes of trainees, to determine if performance is acceptable [2]. In this study, we used the presentation format and parameters for CUSUM scoring used by Kemp et al. [5, 6] Briefly, the CUSUM value was plotted on the y axis against the attempt on the x axis [2, 5, 6]. The CUSUM value is the running sum of a mixture of increments (for each failure) and decrements (for each success). An acceptable success rate is indicated by a declining trend in CUSUM score, whereas a lower-than-expected success rate is indicated by an increasing trend in CUSUM score. For EBUS-TBNA, the designated acceptable failure rate (p0) was 10% and the unacceptable failure rate (p1) was 20%. *s* is the CUSUM score given for each success and is calculated using the equation:

$$ \mathsf{P} = \mathsf{ln}\left(\mathsf{p}\mathsf{1}/\mathsf{p}\mathsf{0}\right)\ \mathsf{Q} = \mathsf{ln}\left(\left(\mathsf{1}\ \hbox{--}\ \mathsf{p}\mathsf{0}\right)/\left(\mathsf{1}\ \hbox{--}\ \mathsf{p}\mathsf{1}\right)\right)\ \mathsf{s} = \mathsf{Q}/\left(\mathsf{P} + \mathsf{Q}\right) $$

Thus:

$$ \mathsf{p}\mathsf{0} = \mathsf{0}.\mathsf{1}\ \mathsf{p}\mathsf{1} = \mathsf{0}.\mathsf{2}\ \mathsf{s} = \mathsf{0}.\mathsf{1}\mathsf{4}\ \mathsf{1}\hbox{--} \mathit{\mathsf{s}} = \mathsf{0}.\mathsf{86} $$

On CUSUM analysis, a trainee was considered “proficient” when the line on the graph descended and crossed 2 boundary lines on a downward slope. A trainee was considered “nonproficient” if the line ascended and crossed 2 boundary lines on an upward slope, which could prompt an intervention such as retraining of the operator. The assessment was considered “inconclusive” if the line remained between the 2 boundary lines. In such cases, additional attempts are necessary in order to determine competence [6, 7]. H0 denotes the value between each acceptable decision interval, and H1 indicates the value between each unacceptable decision level. To determine H, one needs to set the type 1 error (odds of falsely deeming an operator nonproficient, designated as α below) and type 2 error (odds of falsely deeming an operator proficient, designated as β below). We set both to 0.1 in the present analysis. H0 and H1 were calculated as follows:

$$ \mathsf{a} = \mathsf{ln}\left(\left(\mathsf{1}\hbox{--} \mathsf{\beta}\right)/\mathsf{\alpha}\right)\mathsf{b} = \mathsf{ln}\left(\left(\mathsf{1}\ \hbox{--}\ \mathsf{\alpha}\right)/\mathsf{\beta}\right)\mathsf{H}\mathsf{0} = \mathsf{b}/\left(\mathsf{P} + \mathsf{Q}\right)\mathsf{H}\mathsf{1} = \mathsf{a}/\left(\mathsf{P} + \mathsf{Q}\right) $$

Thus, in our series:

$$ \mathsf{H}\mathsf{0} = \mathsf{2}.\mathsf{71}\ \mathsf{H}\mathsf{1} = \mathsf{2}.\mathsf{71} $$

To investigate chronological changes in learning environment, multivariate logistic regression analysis was used to compare each index before and after time points when significant proficiency changes occurred. To evaluate factors associated with a worsening interval, a multivariate-adjusted logistic regression model was constructed by including the presence of additional attending bronchoscopists or on-site cytopathologists, sedative dose (midazolam dose of ≥10 mg vs a lower dose), and opiate dose (fentanyl dose of ≥300 μg vs a lower dose). For measures of association, 95% confidence intervals were computed, and statistical significance was set at a *P* value of <0.05 (2-tailed). STATA 12.0 software was used for all analyses.

## Results

**Fig. 1:**
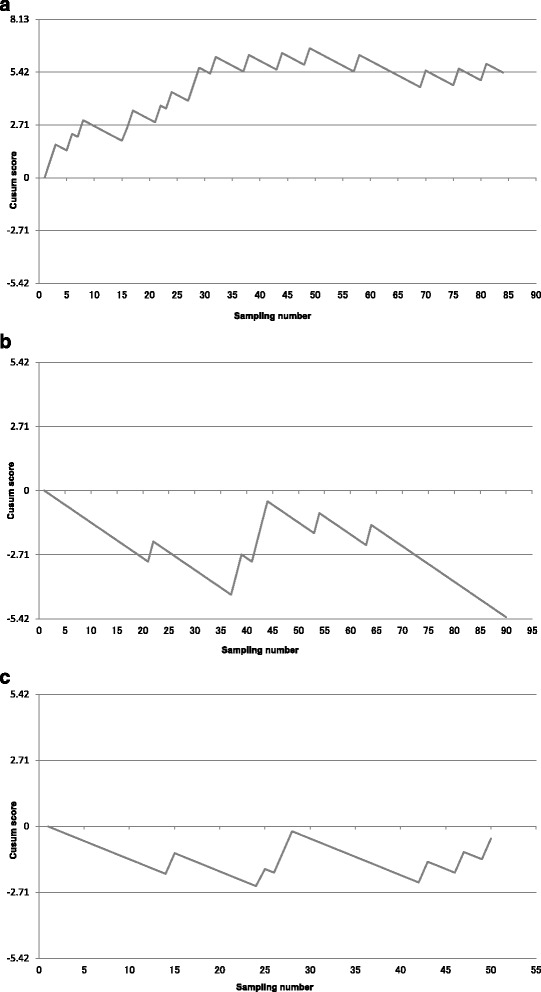
**a** CUSUM Graphs of learning curves for operator A. **b** CUSUM Graphs of learning curves for operator B. **c** CUSUM Graphs of learning curves for operator C

**Fig. 2:**
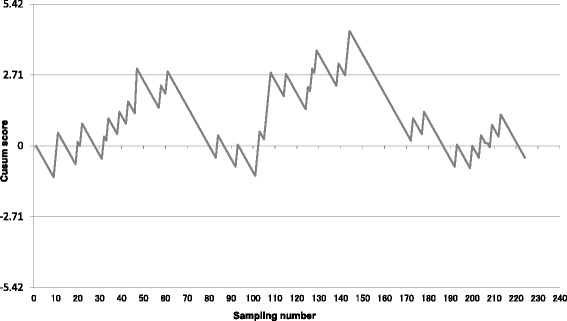
CUSUM Graph of institutional learning curve

Figure 1 shows CUSUM graphs depicting operator learning curves. Failed sampling attempts are indicated by upward deflections in the plot, and successful sampling attempts are indicated by downward deflections. The interval until the line exhibits a stable horizontal or downward direction, without crossing the boundary above it, can be regarded as the time required for an operator to become proficient in EBUS-TBNA. Operator A showed nonproficiency until the 30th sampling attempt, after which he or she exhibited proficiency (Fig. 1). Operator B was proficient from the start of observation until the 36th sampling, after which he or she became nonproficient until the 45th sampling (Fig. 1b). Operator C was proficient throughout the observation period and never crossed the unacceptable boundary (Fig. 1c). Fig. 2 shows the CUSUM learning curve for the institution as a whole. It shows significant chronological changes in proficiency before and after sampling numbers 61, 102, and 142, which correspond to cases 26, 51, and 73, respectively. Institutional proficiency was attained at case 27. However, the success rate started to decline to an unacceptable level after case 51 until proficiency was again attained, starting at case 73.

In the analysis of institutional EBUS-TBNA proficiency, we divided the study period into 4 intervals, namely, the intervals between cases 1 and 26 (learning interval), between cases 27 and 51 (proficiency interval), between cases 52 and 73 (worsening interval), and after case 73 (proficiency interval). To identify factors associated with success rate, we compared indices during the worsening interval (between cases 52 and 73) with those during the other intervals. A multivariate-adjusted logistic regression model was used to analyze the effects of the presence of additional attending bronchoscopists and on-site cytopathologists and higher doses of sedatives (≥10 mg of midazolam) and opiates (≥300 microgram of fentanyl). Comparison of the worsening interval with the other intervals revealed that presence of an additional attending bronchoscopist was inversely associated with nonproficiency (odds ratio [OR], 0.117; 95% confidence interval [CI], 0–0.749; *P* = 0.019). Other factors were not significantly associated with nonproficiency, including presence of on-site cytopathologists (OR, 0.365; 95% CI, 0.0369-1.835; *P* = 0.322), use of a midazolam dose of ≥10 mg (OR, 0.847; 95% CI, 0.167-3.512; *P* = 0.999, *n* = 38), and use of a fentanyl dose of ≥200 μg (OR, 2.786; 95% CI, 0.562-15.471; *P* = 0.2588, *n* = 22). The numbers of procedures performed by operators A and B did not significantly differ in any interval. Operator C performed fewer procedures than the other operators during intervals 1 (*P* < 0.016), 3 (worsening interval) (*P* < 0.045), and 4 (*P* < 0.075).

## Discussion

### Summary of results

A previous study using CUSUM analysis of the EBUS-TBNA learning curve found that the learning rate varied, even among experienced bronchoscopists [5]. In addition, our data suggest that chronological analysis and comparisons of variables before and after time points when significant proficiency changes occurred could be useful in improving the EBUS-TBNA learning environment.

The findings of this pilot study indicate that CUSUM analysis was useful in detecting deviations in bronchoscopist performance and identifying environmental factors associated with such performance. This is the first study to show that CUSUM analysis could be used to investigate the EBUS-TBNA learning environment and identify possible causes for performance declines. We believe that CUSUM analysis can hasten intervention when changes in performance levels are detected.

Both individual and institutional factors affect successful performance of EBUS-TBNA. In this study, we found that presence of additional assisting attending bronchoscopists was associated with successful performance of EBUS-TBNA, which suggests that having an assisting attending bronchoscopist was associated with successful procedures during the institutional learning phase at our center. During the study period, additional attending physicians assisted in identifying target lymph nodes and needle activation. When an assisting attending physician was not present, a respiratory therapist or fellow physician assisted with the procedures. It is possible that the presence of an assisting attending physician resulted in better identification of target lymph nodes, better sampling technique, or both. Operator B exhibited the steepest learning curve, while operator A had the slowest learning curve among the operators. Further quality improvement efforts could attempt to use the present results for detailed investigation and comparison of the learning environments of these 2 operators. For example, operator B might have had greater input from other bronchoscopists. Interestingly, the presence of cytopathologists for rapid on-site evaluation in the bronchoscopy suite did not seem to affect the likelihood of a successful procedure. This finding contradicted our expectations, as we had assumed that the availability of a cytopathologist for rapid on-site evaluation would positively affect performance because lymph node sampling could then be repeated until satisfactory samples were obtained. The change in proficiency during the worsening interval did not appear to coincide with the change in the ratio of procedures performed by the bronchoscopist.

We used new definitions of EBUS-TBNA success and failure. We believe that these definitions are better for determining bronchoscopic proficiency than those used by Kemp et al. [5], as our definitions do not penalize bronchoscopists for the inherent limitations of the FNA procedure.

### Study limitations and implications

This study has several limitations, which are mainly the result of its retrospective design. We were able to obtain information on only a limited number of environmental factors in our bronchoscopy suite. In particular, we lacked data on the technique and performance-based factors. We also had no information on changes in bronchoscopist lifestyle that might have affected performance. Additionally, we had no way of determining the “degree of difficulty” for each attempted sample. The presence of subcentimeter lymph nodes in less accessible locations might decrease success rate, even for a moderately proficient bronchoscopist. To address these limitations and increase the feasibility of this method for quality improvement, our next investigation will be a prospective study. The variables assessed will be more comprehensive, including bronchoscopist work hours, sleep hours, technique and performance-based factors, teaching methods, location and size of lymph nodes, and factors involved in specimen processing.

## Conclusions

Combined CUSUM and environmental analysis is a promising method for monitoring and improving performance in EBUS-TBNA.

## Abbreviations

CUSUM: Cumulative sum
EBUS-TBNA: Endobronchial ultrasound-guided transbronchial needle aspiration
